# Lactoferrin combined with *Bifidobacterium animalis* subsp. *lactis* BB-12 improves respiratory tract infections and modulates Gut microbiome function in children: a randomized, double-blind, placebo-controlled trial

**DOI:** 10.3389/fped.2026.1778240

**Published:** 2026-04-10

**Authors:** Zhe Shi, Huajiang Zhu, Xiaohan Tao, Feitong Liu, Lingling Zhao, Jiahui Zhang, Jinping Zhang

**Affiliations:** 1 Department of Pediatrics, Shanghai Sixth People’s Hospital Affiliated to Shanghai Jiao Tong University School of Medicine, Shanghai, China; 2 Department of Public Health, Shanghai Pudong New Area Shuyuan Community Health Service Center, Shanghai, China; 3 H&H Group, China Research and Innovation Center, Guangzhou, China

**Keywords:** bifidobacterium, gut microbiome, host–microbe interactions, microbiome-metabolome interactions, respiratory tract infections

## Abstract

**Objectives and study:**

To explore the effects and mechanisms of lactoferrin combined with *Bifidobacterium animalis* subsp. *lactis* BB-12 on Respiratory tract infections (RTIs) in healthy children through a randomized, double-blind, placebo-controlled trial.

**Methods:**

Eligible healthy children aged 0–6 years were randomized into intervention group (IG, *n* = 60, receiving lactoferrin and probiotic for 3 months) and control group (CG, *n* = 34, receiving placebo). The study primarily assessed changes in the incidence and severity of RTIs, and monitored gastrointestinal adverse events. Fecal samples were collected pre- and post-intervention to analyze gut microbiota composition and metabolomic profiles, including short-chain fatty acids (SCFAs).

**Results:**

After the intervention, the incidence of RTIs showed no significant difference between groups (55% vs. 61.8%, *p* = 0.52), but both the RTI severity score (3.0 vs. 4.0, *p* < 0.05) and mean duration per RTI episode (3.54 d vs. 5.27 d, *p* < 0.05) were significantly lower in the IG. No serious adverse events were reported, and the incidence of indigestion was significantly reduced in the IG compared with the CG (8.3% vs. 23.5%, *p* = 0.04). The intervention significantly altered phylogenetic diversity (PD-whole tree within IG: *p* = 0.0011; baseline between IG and CG: *p* = 0.63; post-intervention between IG and CG: *p* = 0.029) and community structure (weighted UniFrac within IG: 0.012; between IG and CG at 3 months: *p* = 0.036 vs. baseline *p* = 0.01). The gut microbiota in the intervention group exhibited a trend toward greater stability over time. Integrated microbiome–metabolite analysis showed attenuation of fatty acid oxidation–and energy metabolism-related metabolic drivers after intervention, together with no significant changes in fecal SCFA levels.

**Conclusions:**

The intervention improved clinical outcomes and induced phylogenetic restructuring of the gut microbiota rather than changes in overall abundance, accompanied by a shift toward greater stability in gut microbial structure and energy metabolic patterns.

**Clinical Trial Registration:**

identifier ChiCTR2500111308.

## 1 Introduction

Respiratory tract infections (RTIs) are among the most prevalent infectious conditions in childhood. They typically result from viral or bacterial invasion of the respiratory tract and represent a major contributor to antibiotic overuse and the escalating burden of antimicrobial resistance. Owing to the immaturity of the immune system and mucociliary clearance, children are particularly susceptible to recurrent RTIs.

At present, preventive strategies for RTIs rely largely on vaccination and pharmacological interventions; however, RTIs are caused by a wide spectrum of pathogens, limiting the availability of effective and broadly protective vaccines. Immunotherapeutic approaches have therefore been proposed as a means to reduce infection risk by modulating host immune defenses. Although numerous interventions, including vitamins, trace elements, and bioactive polysaccharides, have been explored, sustained and reliable protection against RTIs in susceptible children remains elusive. To date, the largest body of clinical evidence supporting both efficacy and safety exists for bacterial lysate vaccines, although heterogeneity among studies and limitations in study quality temper the strength of recommendations (1, 2).

Evidence to date suggests that probiotics may reduce the incidence and severity of RTIs via immunomodulatory mechanisms and reinforcement of mucosal barrier function (3). Despite this, the current evidence base is characterized by substantial strain heterogeneity, limited sample sizes, inconsistent intervention duration and dosing, and a focus on specific populations.

The present study was designed to address these limitations by providing additional clinical evidence, with the aim of supporting the clinical applicability and generalizability of probiotics in reducing the burden of RTIs. In this context, a combined formulation of lactoferrin and *Bifidobacterium animalis* subsp. *lactis* BB-12 was evaluated. *Bifidobacterium animalis* subsp. *lactis* BB-12, which served as the core probiotic component of the intervention in the present study, has been shown to modulate gut microbiota composition, enhance mucosal immune function, and reduce infection susceptibility (4, 5).

Lactoferrin is a component of the innate immune system with documented antibacterial, antiviral, and immunomodulatory effects. Through iron chelation, it inhibits pathogen growth, promotes mucosal epithelial repair, and modulates the function of multiple immune cell populations. Previous studies have further suggested that lactoferrin may reduce the incidence of RTIs and shorten disease duration, with particularly pronounced effects in infants and immunologically vulnerable populations (6).

Against this background, a potential synergistic interaction between lactoferrin and probiotics has been proposed. Lactoferrin may support probiotic colonization and functional activity by optimizing the intestinal mucosal environment and enhancing local immune responses (7). Conversely, probiotics may facilitate the proteolytic processing of lactoferrin into bioactive peptides, potentially enhancing its antimicrobial activity (8). The combined intervention may exert complementary effects on mucosal immune regulation, suppression of opportunistic pathogen expansion, and maintenance of gut microbiota homeostasis, thereby providing greater protection against RTIs than either component alone. Based on this theoretical framework, we evaluated the preventive effects of this combined formulation against RTIs in children in a randomized controlled trial.

## 2 Methods

### 2.1 Study design and ethical approval

This randomized, double-blind, placebo-controlled clinical trial was conducted in accordance with the Declaration of Helsinki, ICH-GCP standards, and CONSORT 2025 reporting guidelines, and comprised a 3-month intervention period followed by a 1-month follow-up. Participants were randomly assigned in a 2:1 ratio to receive either the intervention or placebo according to a computer-generated randomization schedule prepared by an independent statistician. Allocation concealment was ensured using identical packaging, and both participants and investigators were blinded to group assignment until completion of data analysis.

The study protocol was approved by the Human Ethics Committee of Shanghai Sixth People's Hospital [Approval No. 2025-039-(1)] and registered at the Chinese Clinical Trial Registry (ChiCTR2500111308). Written informed consent was obtained from parents or legal guardians prior to enrollment.

The study was conducted in a hospital setting at the Department of Pediatrics, Shanghai Sixth People's Hospital Affiliated to Shanghai Jiao Tong University School of Medicine, Shanghai, China.

### 2.2 Participants

Participants were eligible for inclusion if they met the following criteria: (1) healthy children; (2) currently receiving exclusive formula feeding or not consuming infant formula; (3) aged 0–6 years; and (4) having experienced at least one episode of RTI within the past year. Participants were excluded if any of the following criteria were present: (1) current breastfeeding or mixed feeding (breast milk combined with formula); (2) exclusive formula feeding with formulas containing lactoferrin and/or *Bifidobacterium animalis* subsp. *lactis* BB-12; (3) ongoing use of probiotic supplements or lactoferrin-containing products; (4) a history of severe infection or having undergone surgical procedures; or (5) a personal or family history of milk allergy or lactose intolerance.

### 2.3 Intervention

The investigational product consisted primarily of *Bifidobacterium animalis* subsp. *lactis* BB-12 (2 × 10^9^ CFU per sachet) and lactoferrin (18 mg per sachet), together with skimmed milk powder, whey protein powder (including whey protein concentrate and whey protein isolate), whole milk powder, and galacto-oligosaccharides. The product was provided by Biostime (Guangzhou) Health Products Ltd. (Guangzhou, China), with the production batch number 420139002.

Participants in the intervention group received one sachet per dose, administered twice daily. The product was dissolved in warm water or mixed with semi-liquid foods such as rice cereal prior to consumption. The intervention period lasted for 3 months. The placebo group received identical sachets containing excipients only, which consisted of skimmed milk powder, whey protein powder (including whey protein concentrate and whey protein isolate), whole milk powder, and galacto-oligosaccharides.

### 2.4 Blinding

After random assignment, participants, caregivers, investigators, outcome assessors, and data analysts were blinded to group allocation. Blinding was achieved by using interventions and placebos that were identical in appearance, packaging, taste, and labeling. The randomization code was not broken until completion of data analysis.

### 2.5 Outcome measures

The primary outcome was the frequency of RTI episodes during the 3-month intervention period, compared between the intervention and placebo groups, as recorded through monthly structured caregiver-completed questionnaires.

Secondary outcomes included RTI symptom severity and duration, gastrointestinal symptom burden, and gut microbiome-related outcomes. RTI symptoms (e.g., cough, fever, and nasal discharge) and gastrointestinal discomfort were assessed monthly, with symptom-related outcomes analyzed using cumulative data collected over the entire study period. Symptom severity was quantified using a 0–10 numerical rating scale, where 0 indicated no symptom and 10 indicated the most severe symptom. According to predefined criteria, symptom severity scores were further categorized into three levels: mild (0–3), moderate (4–7), and severe (8–10). These severity levels were used to evaluate and compare the symptom burden across both groups. Changes in gut microbiota composition and metabolomic profiles, including short-chain fatty acids (SCFAs), were also evaluated to explore potential microbiome-mediated mechanisms underlying the intervention effects. Comparisons of gut microbiota diversity, taxonomic composition, and metabolomic profiles were conducted between baseline (0 days) and the end of the intervention (3 months). Longitudinal analyses further incorporated data from baseline (0 days), day 21, and 3 months to characterize intervention-associated metabolic trends.

During the study, in the event of a respiratory tract infection, participants will receive treatment according to standard clinical protocols, including antibiotics and symptomatic medications such as acetaminophen and chlorpheniramine, as deemed appropriate.

### 2.6 Assessment and recording of adverse events

Adverse events were defined as any unfavorable medical occurrences reported after the administration of the investigational product during the clinical trial, regardless of whether a causal relationship with the intervention was established. Serious adverse events were defined as events that resulted in death, were life-threatening, required hospitalization or prolonged existing hospitalization, caused significant or persistent disability or impairment of functional capacity, or led to congenital anomalies or birth defects.

In this study, adverse events were systematically monitored throughout the intervention and follow-up periods. At each study visit and during follow-up, caregivers were asked to complete structured questionnaires that included specific items related to potential adverse reactions. In addition, investigators actively inquired about adverse events and recorded all reported events in the case report forms.

### 2.7 Sample size

The sample size was determined based on evidence from previously published randomized controlled trials evaluating lactoferrin and probiotic interventions for the prevention of RTIs. In the general pediatric population, children experience an average of five RTI episodes per year (9). Assuming a 25% reduction in RTI incidence with the intervention (10), with a two-sided significance level of *α* = 0.05 and a statistical power of 80% (1 − *β* = 0.80), the minimum required sample size was estimated to be 90 participants. Allowing for an anticipated dropout rate of approximately 10%, a total of 99 participants were planned for enrollment.

### 2.8 Statistical analysis

All clinical data were analyzed using GraphPad Prism (version 10.0; GraphPad Software, San Diego, CA, USA). Continuous variables were assessed for normality using the Shapiro–Wilk test. Normally distributed data are presented as mean ± standard deviation (SD) and compared between groups using the independent-samples *t*-test. Non-normally distributed data are presented as median (interquartile range, IQR) and analyzed using the Mann–Whitney *U*-test. Categorical variables are expressed as frequencies and percentages and compared using the *χ*^2^ test; Fisher's exact test was applied when expected cell counts were <5. Missing data were not imputed.

### 2.9 Gut microbiome analysis

Gut microbiome sequencing data were analyzed using the QIIME2 platform (version 2023.2). Raw sequences were subjected to quality control, denoising, and merging to generate amplicon sequence variants (ASVs), which were taxonomically annotated against the SILVA database (version 138).

Alpha diversity was assessed using ACE, Chao1, Good's coverage, Observed species, Shannon, Simpson, and PD-whole tree indices to evaluate within-sample diversity. Between-group differences in alpha diversity indices were assessed using the Wilcoxon rank-sum test. Beta diversity was evaluated based on weighted UniFrac, unweighted UniFrac, and Bray–Curtis distance matrices to compare community composition between groups. Between-group differences in beta diversity were assessed using permutational multivariate analysis of variance (PERMANOVA) and analysis of similarities (ANOSIM).

Differences in relative abundance at various taxonomic levels were initially screened using the Kruskal–Wallis test and further analyzed and visualized using the Statistical Analysis of Metagenomic Profiles (STAMP) software. Linear Discriminant Analysis Effect Size (LEfSe) analysis was subsequently applied to identify discriminative taxa, with significance thresholds set at *P* < 0.05 and an LDA score > 2.

### 2.10 Metabolomic analysis

Untargeted metabolomic analysis of fecal samples was performed using an ultra-high-performance liquid chromatography–tandem mass spectrometry (UHPLC–MS/MS) platform. Samples were prepared following standard procedures, and quality control (QC) samples were included throughout the analytical run to monitor instrument stability and data reproducibility.

Raw mass spectrometry data were processed for peak detection, alignment, and quantification. Metabolite annotation was performed based on accurate mass, retention time, and MS/MS spectral information. Data were normalized and log-transformed prior to downstream analyses to improve comparability across samples.

Differential metabolites were identified based on between-group comparisons and visualized using volcano plots. Subsequently, pathway enrichment analysis of differential metabolites was conducted based on the Kyoto Encyclopedia of Genes and Genomes (KEGG) database to explore intervention-associated metabolic pathway alterations.

### 2.11 Integrated analysis

Using the genus-level differentially abundant taxa (identified by LEfSe) and significantly altered metabolites, two data matrices were subjected to integrated microbiome–metabolome analysis: the matrix of all samples at 3 months (cross-sectional), and the matrix of paired delta values (change from baseline) within each group (longitudinal). Spearman rank correlation analysis was used to evaluate associations between differential taxa and differential metabolites. Taxa–metabolite pairs with an absolute correlation coefficient ≥0.5 and *P* < 0.05 were used to construct a Spearman correlation network.

Associations between overall gut microbial community structure and metabolomic profiles were further assessed using the Mantel test. Redundancy analysis (RDA) was subsequently conducted to explore the extent to which differential metabolites explained variations in microbial community composition. Environmental variables included in the RDA were defined as the top 10 differentially abundant metabolites with qualitative annotation, ranked by VIP values and meeting *P* < 0.05; when fewer than 10 metabolites met these criteria, all eligible metabolites were included in the analysis.

## 3 Results

**Figure 1:**
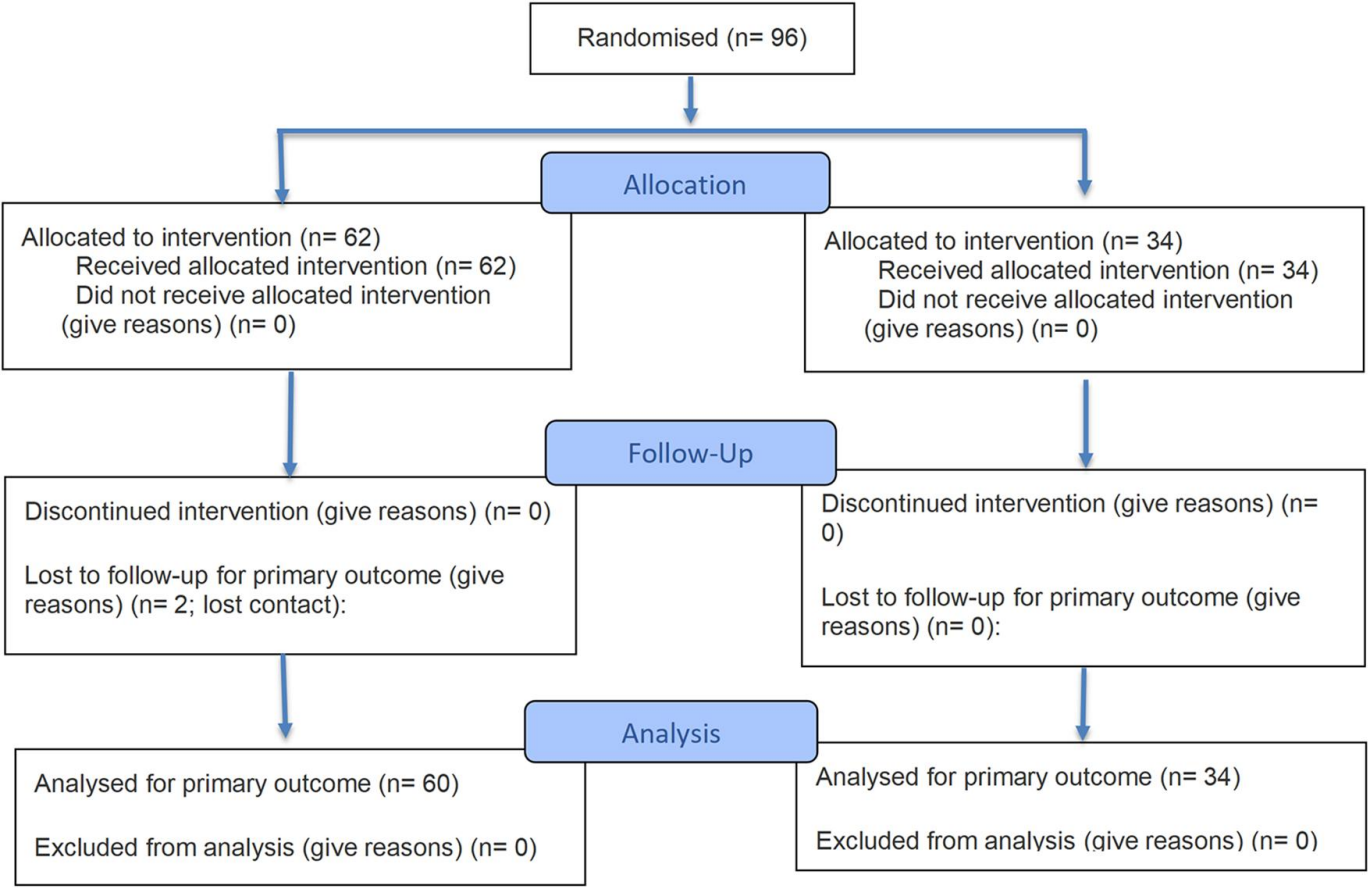
CONSORT flow diagram of the trial.

The study was conducted between May 9 and October 23, 2025. The intervention and placebo were administered as intended throughout the study period, with good participant adherence. No concomitant care was received during the trial in either group. Participant flow through the trial is shown in Figure 1.

### 3.1 Baseline characteristics

The study included 94 children (control group: *n* = 34; intervention group: *n* = 60). No significant differences were observed between groups in baseline demographic or clinical characteristics, including sex distribution, age, mode of delivery, feeding pattern during the first 6 months, and the incidence of respiratory infections within the preceding 21 days (all *P* > 0.05; Table 1).

**Table 1:** Baseline characteristics of enrolled participants.

Variable	Total	Control	Treated	*P*
Number	94	34	60	
Male, *n* (%)	52 (55.3)	22 (64.7)	30 (50.0)	0.245
Age, months	51.1 ± 23.2	49.2 ± 22.5	52.2 ± 23.7	0.732
Aged ≥3 years, *n* (%)	69 (73.4)	24 (70.6)	45 (75.0)	0.642
Drug use. *n* (%)	2 (2.1)	1 (2.9)	1 (1.7)	0.595
Caesarean, *n* (%)	48 (51.1)	17 (50)	31 (51.7)	1
Feeding Pattern (0–6 Months)				0.624
Full breast feeding, *n* (%)	41 (43.6)	17 (50.0)	24 (40.0)	
Full formula feeding, *n* (%)	20 (21.3)	6 (17.6)	14 (23.3)	
Mix feeding, *n* (%)	33 (35.1)	11 (32.4)	22 (36.7)	
Respiratory infections in the past 21 days, *n* (%)	29 (30.9)	11 (32.4)	18 (30)	0.996

### 3.2 Clinical outcomes

#### 3.2.1 Respiratory symptom outcomes

The incidence of RTIs was 55.0% (33/60) in the intervention group and 61.8% (21/34) in the control group (Table 2). The relative risk was 0.89 (95% CI 0.63–1.30), with an absolute risk difference of 6.8% (95% CI −13.7% to 28.9%). The difference was not statistically significant (Fisher's exact test, *P* = 0.6646).

**Table 2:** Incidence of common cold and medication use.

Outcome	Intervention period			Follow-up period		
	Control (*n* = 34)	Treated (*n* = 60)	*P*	Control (*n* = 34)	Treated (*n* = 60)	*P*
Incidence of common cold, *n* (%)	21 (61.8)	33 (55.0)	0.5239	6 (13.3)	8 (17.7)	0.5633
Drug use, *n* (%)	18 (52.9)	22 (36.7)	0.4206	7 (20.6)	4 (6.7)	0.0905

**Figure 2:**
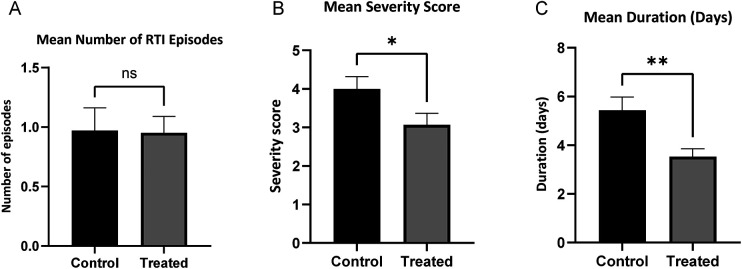
Occurrence of respiratory tract infections (RTIs) during the intervention period (3 months). **(A)** Mean number of RTI episodes; **(B)** mean severity score of RTIs; **(C)** mean duration of RTIs (days). Bars represent means, and error bars indicate the standard error of the mean (SEM). Statistical significance is denoted as: ns, not significant; *P < 0.05; **P < 0.01. The mean number of episodes was calculated based on the total study population, whereas mean severity scores and mean duration were calculated based on RTI episodes.

**Figure 3:**
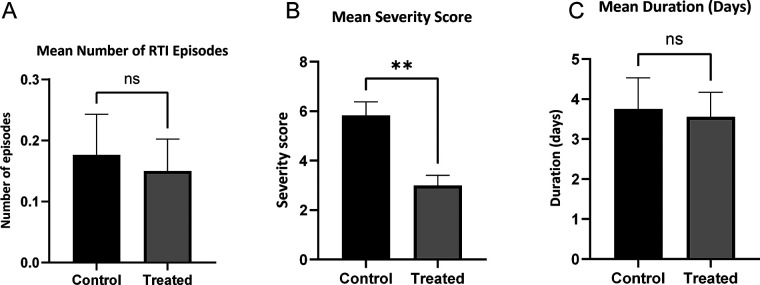
Occurrence of RTIs during the follow-up period (1 month). **(A)** Mean number of RTI episodes; **(B)** mean severity score of RTIs; **(C)** mean duration of RTIs (days). Bars represent means, and error bars indicate the standard error of the mean (SEM). Statistical significance is denoted as: ns, not significant; *P < 0.05; **P < 0.01.

However, the intervention group experienced significantly reduced symptom severity and shorter episode duration (*P* < 0.05; Figure 2). This reduction in symptom severity persisted during follow-up (severity score: 3.13 vs. 5.83, *P* < 0.05), while no between-group differences were observed in RTI incidence or duration (Figure 3).

At the individual symptom level, no significant between-group differences were observed in the incidence, duration, or severity of RTI-related symptoms (Tables 3, 4), with the exception of nasal congestion severity during the intervention period, which was significantly lower in the intervention group (severity score: 3.0 vs. 5.5, *P* = 0.0085; Table 4).

**Table 3:** Incidence of symptoms during the intervention and follow-up periods.

Symptom	Intervention period			Follow-up period		
	Control (*n* = 34)	Treated (*n* = 60)	*P*	Control (*n* = 34)	Treated (*n* = 60)	*P*
Nasal congestion, *n* (%)	15 (44.1)	20 (33.3)	0.2987	2 (5.9)	5 (8.3)	>0.9999
Rhinorrhea, *n* (%)	16（47.1）	26 (43.3)	0.7270	3 (8.2)	4 (6.7)	0.7007
Cough (productive), *n* (%)	10 (29.4)	15 (25.0)	0.6418	2 (5.9)	1 (1.7)	0.2958
Cough (non-productive), *n* (%)	10 (29.4)	10 (16.7)	0.1469	4 (11.8)	3 (5.0)	0.2493
Throat, *n* (%)	9 (26.5)	8 (13.3)	0.1118	3 (8.8)	3 (5.0)	0.6640
Wheezing, *n* (%)	2 (5.9)	4 (6.7)	0.8812	2 (5.9)	1 (1.7)	0.2958
Fever, *n* (%)	8 (23.5)	13 (24.7)	0.8350	5(14.7)	2(3.3)	0.0943

**Table 4:** Mean cumulative duration and severity of symptoms during the intervention period.

Symptom	Intervention period		
	Control (*n* = 34)	Treated (*n* = 60)	*P*
Total days with symptoms, M(P_25_, P_75_)			
Nasal congestion	5.5 [2.0,5.5]	5.5 [2.0,5.9]	0.7701
Rhinorrhea	5.5 [2.0,7.0]	2.0 [2.0,6.4]	0.6390
Cough (productive)	5.5 [2.0,8.9]	2.0 [2.0,5.5]	0.1855
Cough (non-productive)	5.5 [2.0,6.4]	4.8 [2.0,5.5]	0.6405
Throat	4.0 [2.0,6.0]	4.8 [2.5,7.5]	0.5490
Wheezing	5.5 [5.5,5.5]	2.0 [2.0,3.5]	0.0667
Fever	2.0 [2.0,5.0]	2.0 [2.0,4.8]	>0.999
Severity score, M(P_25_, P_75_)			
Nasal congestion	5.5 [2.8,7.3]	3.0 [2.0,4.0]	0.0085
Rhinorrhea	3.0 [3.0,5.0]	3.0 [2.0,5.0]	0.1190
Cough (productive)	3.0 [3.0,5.0]	3.0 [1.0,5.0]	0.5334
Cough (non-productive)	3.0 [2.0,5.0]	2.5 [1.0,4.0]	0.2869
Throat	3.0 [2.0,4.0]	3.0 [2.0,6.0]	0.4594
Wheezing	5.5 [4.0,7.0]	2.0 [1.0,4.5]	0.1429
Fever	5.5 [2.6,7.3]	5.0 [3.0,7.0]	0.5413

During the study, the medication usage rate in the intervention group was 0.366 (22/60), with 1 participant using antibiotics and the remaining 21 using symptomatic cold medications. In the control group, the medication usage rate was 0.529 (18/34), with 5 participants using antibiotics and the remaining 13 using symptomatic medications. However, no statistically significant difference was observed between the medication usage rates in the two groups.

#### 3.2.2 Gastrointestinal symptom outcomes

Across the entire study period, the overall incidence of gastrointestinal discomfort symptoms—including constipation, diarrhea, and indigestion—was lower in the intervention group than in the control group, although the difference was not statistically significant. At the individual symptom level, indigestion occurred in 5 of 60 children (8.3%) in the intervention group and in 8 of 34 children (23.5%) in the control group during the intervention period (Table 5). The relative risk was 0.35 (95% CI 0.13–0.96), corresponding to an absolute risk difference of 15.2% (95% CI −3.6% to 31.4%). The difference was statistically significant according to the chi-square test (*P* = 0.0403).

**Table 5:** Incidence of gastrointestinal discomfort symptoms during the intervention and follow-up periods.

Outcome	Intervention period			Follow-up period		
	Control (*n* = 34)	Treated (*n* = 60)	*P*	Control (*n* = 34)	Treated (*n* = 60)	*P*
Incidence of gastrointestinal tract symptoms, *n* (%)	13 (38.2)	14 (28.3)	0.1250	2 (5.9)	5 (8.3)	>0.9999
Constipation, *n* (%)	2 (5.9)	4 (6.7)	>0.9999	1 (2.9)	2 (3.3)	>0.9999
Diarrhea, *n* (%)	3 (8.8)	2 (3.3)	0.3482	0 (0.0)	2 (3.3)	0.5333
Indigestion, *n* (%)	8 (23.5)	5 (8.3)	0.0403	1 (2.9)	1 (1.7)	>0.9999

#### 3.2.3 Adverse events

No adverse events were reported in either group during the study period.

### 3.3 Gut microbiota analysis

#### 3.3.1 Alpha diversity

**Figure 4:**
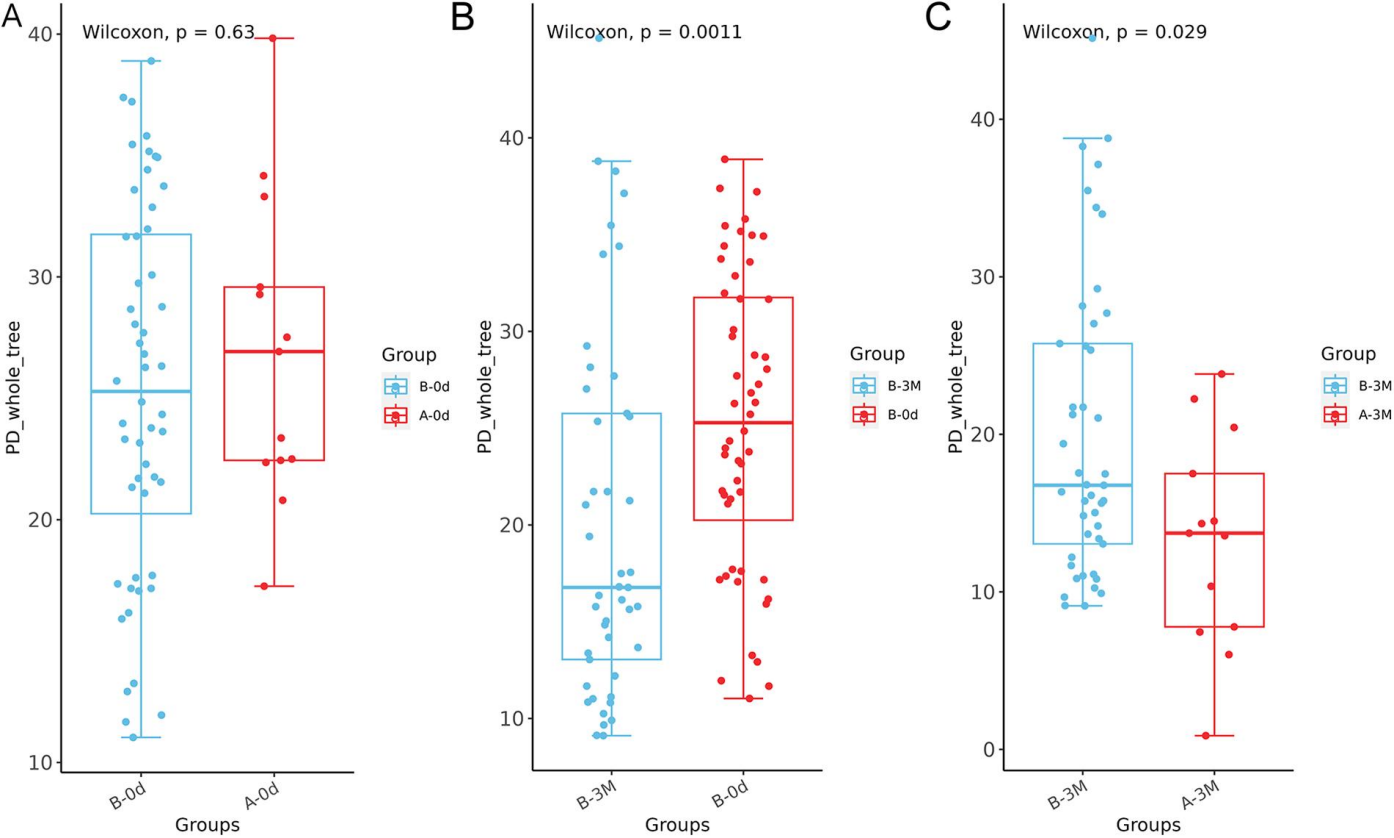
Changes in phylogenetic diversity (PD-whole tree) during the intervention. **(A)** Baseline comparison of PD-whole tree between Group A (control group) and Group B (intervention group). **(B)** Within-group comparison of PD-whole tree in Group B (intervention group) between baseline and 3 months. **(C)** Comparison of PD-whole tree between Group A (control group) and Group B (intervention group) at 3 months.

PD whole tree showed a significant between-group difference at 3 months (*P* = 0.029; Figure 4B) and a significant within-group change from baseline to 3 months in the intervention group (*P* = 0.0011; Figure 4C). No significant differences were observed for the other alpha diversity indices (all *P* > 0.05). Together, these results indicate a shift in phylogenetic diversity rather than a broad change in species richness or evenness.

#### 3.3.2 Beta diversity

Analyses based on Bray–Curtis and unweighted UniFrac distances revealed no significant differences between the intervention and control groups at 3 months, indicating that the intervention did not markedly alter overall species abundance or presence/absence profiles.

**Figure 5:**
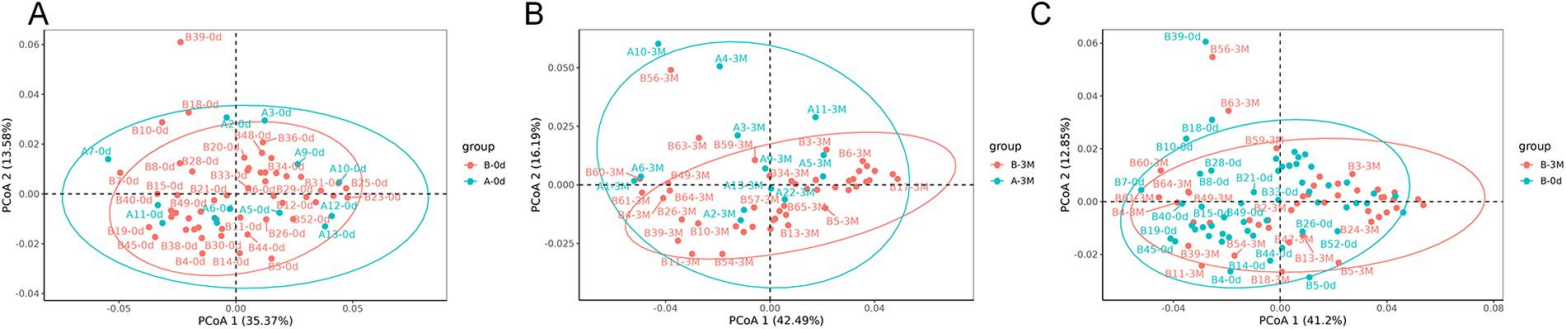
Principal coordinates analysis (PCoA) of gut microbiota community structure based on weighted uniFrac distance. **(A)** Baseline comparison of gut microbiota community structure between Group A (control group) and Group B (intervention group). **(B)** Comparison of gut microbiota community structure between Group A (control group) and Group B (intervention group) at 3 months. **(C)** Within-group comparison of gut microbiota community structure in Group B (intervention group) between baseline and 3 months. Each point represents an individual sample, and colors indicate different groups. Ellipses represent the 95% confidence interval for each group.

In contrast, weighted UniFrac analysis demonstrated clear structural shifts. Principal coordinates analysis (PCoA) showed separation between groups at 3 months (Figure 5B) and within the intervention group over time (Figure 5C). These patterns were statistically supported: significant differences were detected both between groups at 3 months (PERMANOVA: *R*^2^ = 0.045, *P* = 0.036; ANOSIM: *R* = 0.144, *P* = 0.038) and within the intervention group from baseline to 3 months (PERMANOVA: *R*^2^ = 0.035, *P* = 0.012; ANOSIM: *R* = 0.057, *P* = 0.009).

Together, these results demonstrate differences in abundance-weighted phylogenetic structure between groups, without substantial changes in community membership or overall abundance profiles.

#### 3.3.3 Differential taxonomic composition

**Figure 6:**
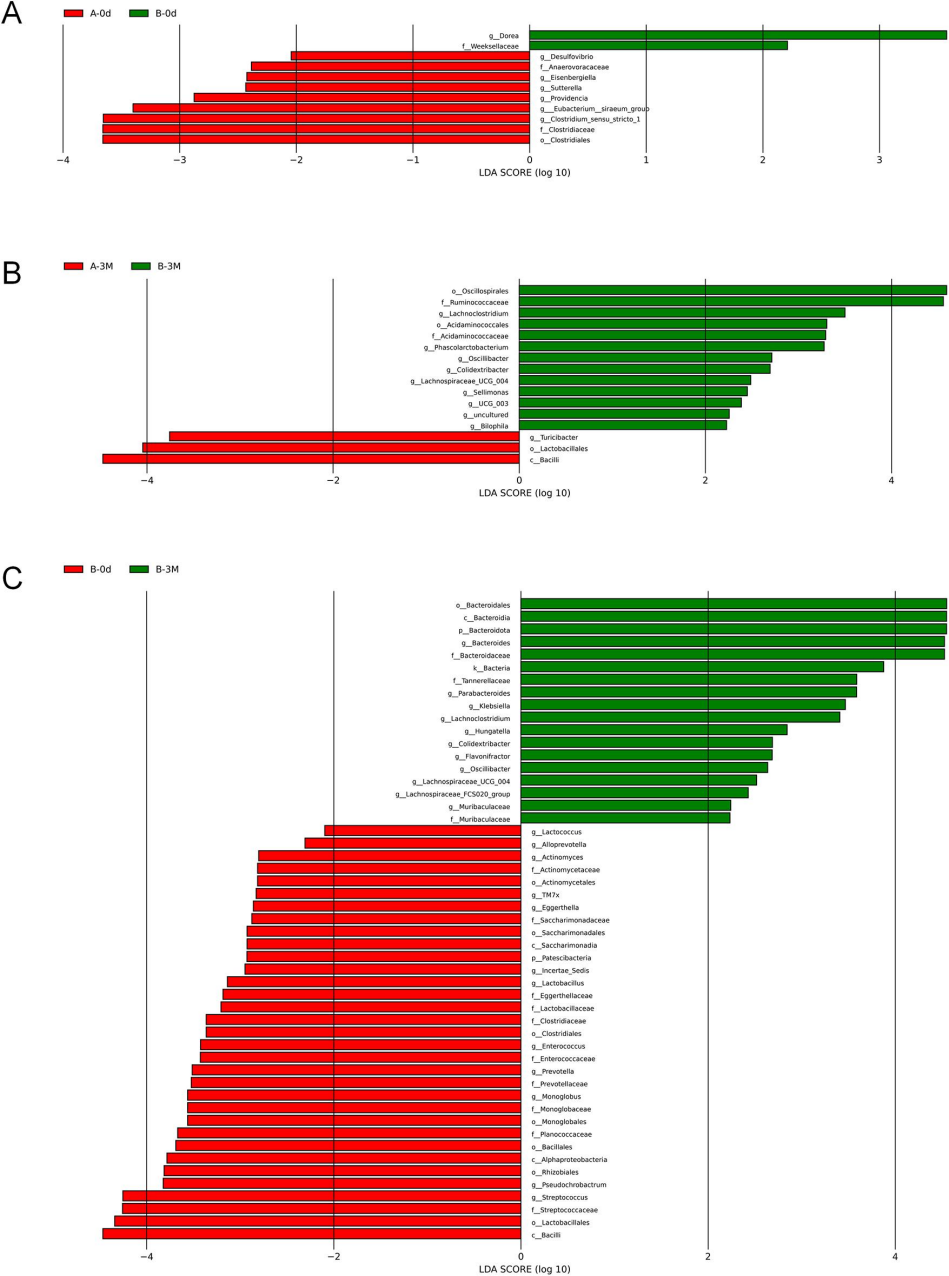
Differentially abundant taxa identified by LEfSe analysis. **(A)** Baseline comparison of taxa composition between Group A (control group) and Group B (intervention group). **(B)** Comparison of taxa composition between Group A (control group) and Group B (intervention group) at 3 months. **(C)** Within-group comparison of taxa composition in Group B (intervention group) between baseline and 3 months.

Differential taxonomic analysis demonstrated baseline differences in gut microbiota composition between the intervention and control groups. LEfSe analysis further indicated that these differences were predominantly driven by pathogenic-associated taxa rather than core beneficial genera (Figure 6).

**Figure 7:**
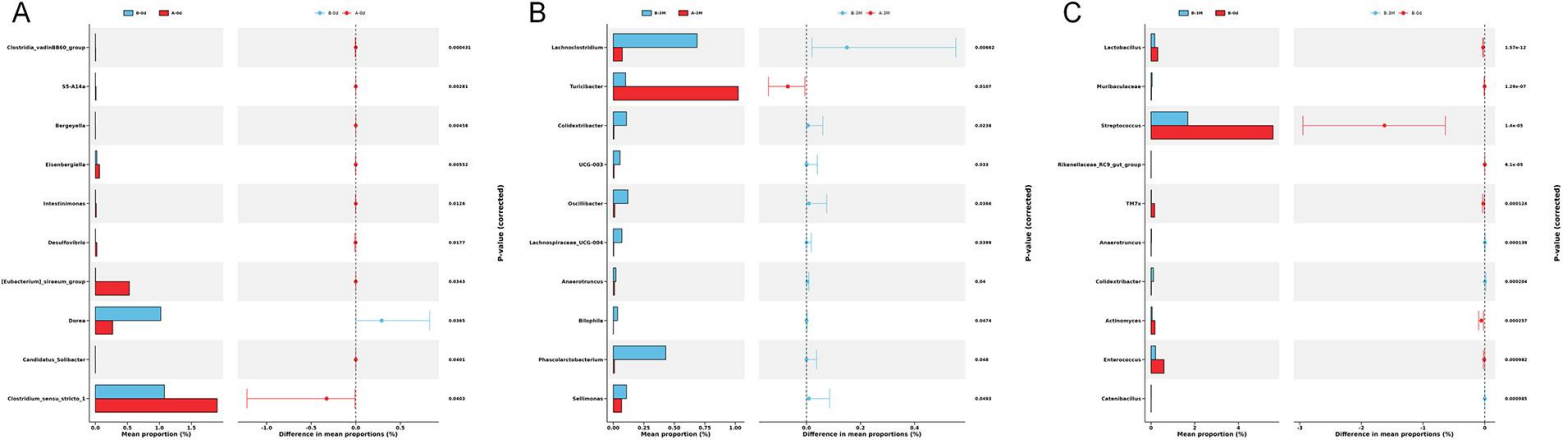
Differentially abundant genera identified by STAMP analysis. **(A)** Baseline comparison of genera between Group A (control group) and Group B (intervention group). **(B)** Comparison of genera between Group A (control group) and Group B (intervention group) at 3 months. **(C)** Within-group comparison of genera in Group B (intervention group) between baseline and 3 months.

Following the intervention, genus-level analyses revealed more consistent and directionally aligned changes. LEfSe analysis identified several discriminative genera enriched in the intervention group, including *Lachnoclostridium* and *Oscillibacter* (LDA score > 2), which are commonly associated with SCFAs production and amino acid metabolism, whereas the control group was enriched in *Turicibacter*, a genus related to potential pathogenicity (Figure 6). These findings were further supported by STAMP analysis, which quantitatively confirmed consistent between-group differences in relative abundance (all *P* < 0.05; Figure 7).

**Figure 8:**
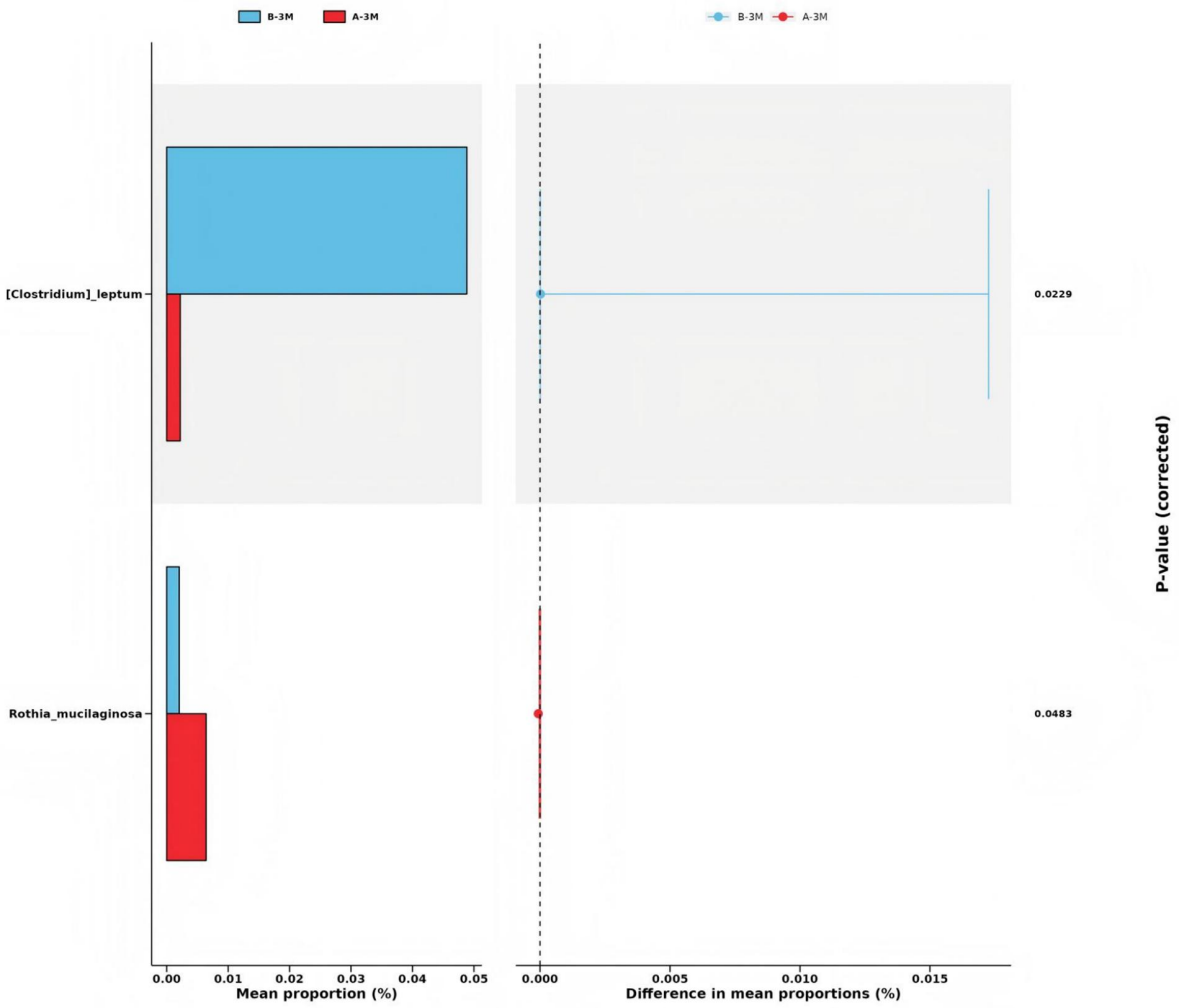
Differentially abundant species between group A (control group) and group B (intervention group) at 3 months identified by STAMP analysis.

At a finer taxonomic resolution, Taxa affiliated with the *Clostridium leptum* group were significantly enriched in the intervention group, whereas *Rothia mucilaginosa*, a species frequently linked to inflammatory or dysbiotic states, was more abundant in the control group (all *P* < 0.05; Figure 8).

#### 3.3.4 Longitudinal shift in Gut microbiota composition

**Figure 9:**
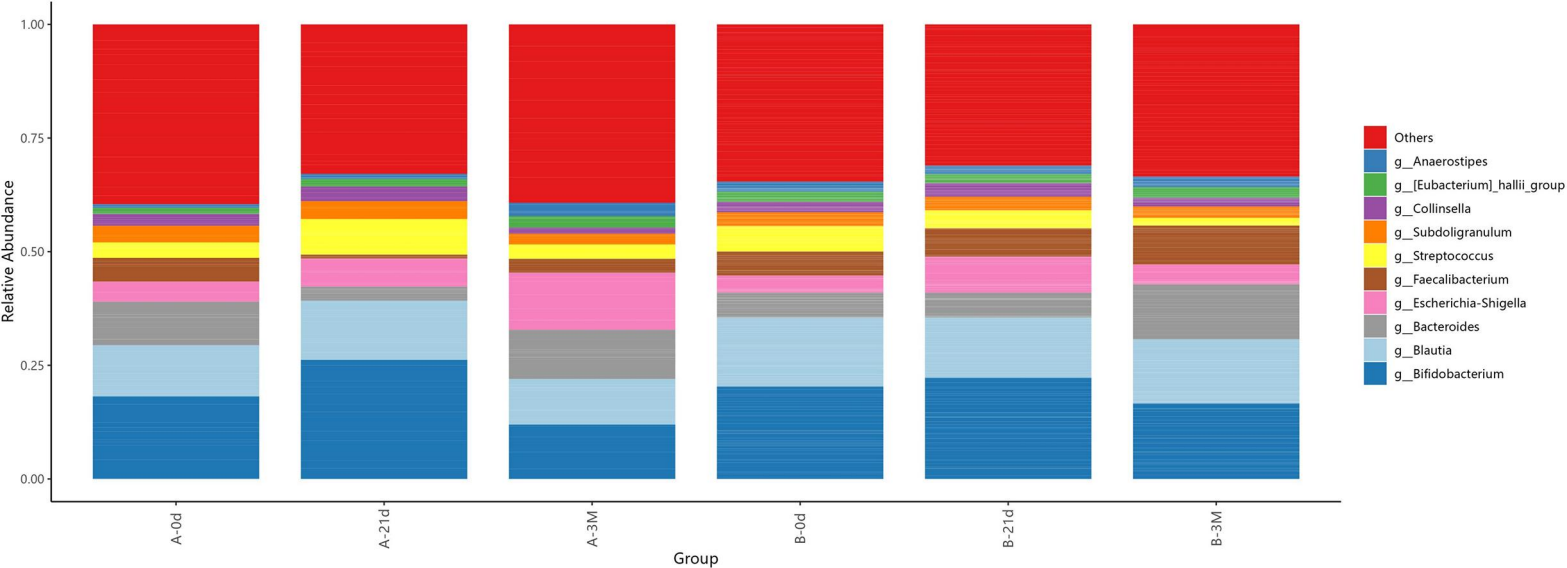
Genus-level relative abundance of gut microbiota across groups and time points.

Longitudinal analysis revealed distinct temporal dynamics in gut microbiota composition between groups (Figure 9). The overall community structure in the intervention group exhibited relatively limited variation over time, whereas the control group showed more pronounced compositional changes across time points.

The relative abundance of *Bifidobacterium* showed an early increase followed by a moderate decline at 3 months, while *Bacteroides* exhibited a sustained increase; the combined abundance of these two taxa remained higher in the intervention group than in the control group. *Faecalibacterium* increased progressively over time in the intervention group but declined sharply in the control group at day 21, with only partial recovery thereafter.

In contrast, *Escherichia–Shigella* displayed a continuous increasing trend in the control group, while remaining relatively stable in the intervention group.

### 3.4 Metabolomic analysis

#### 3.4.1 Fecal short-chain fatty acid profiles

**Figure 10:**
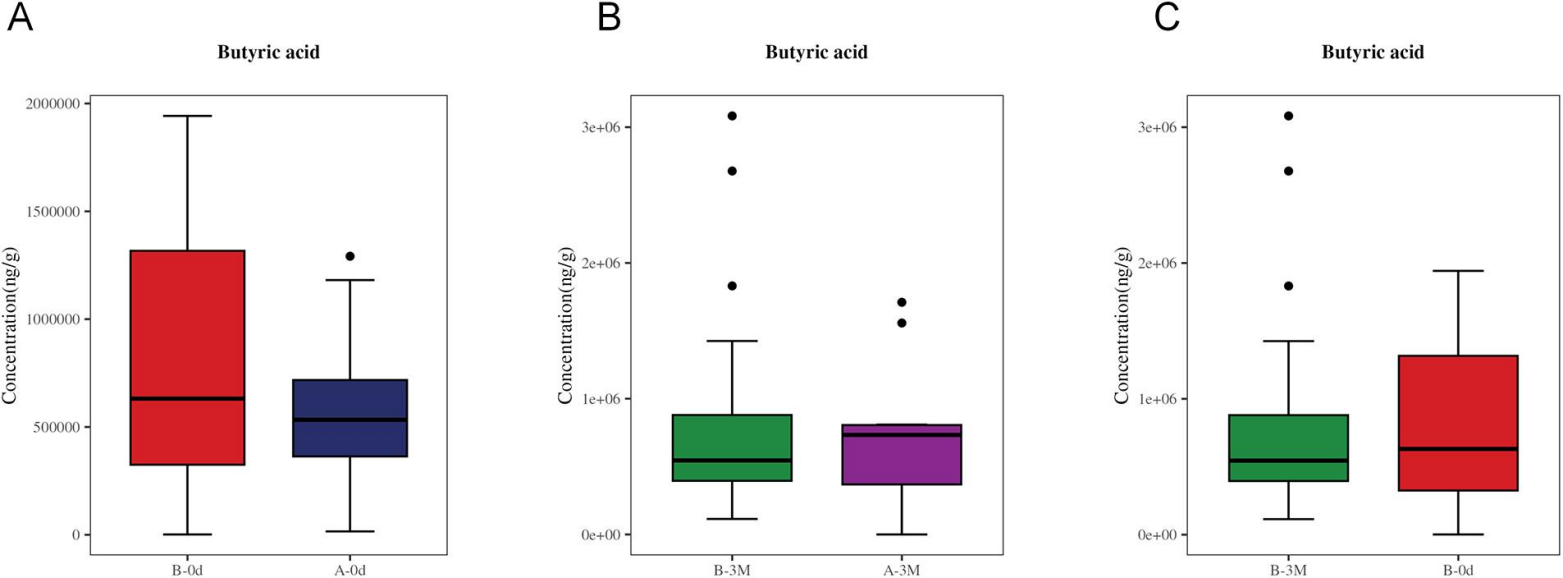
Comparison of fecal butyric acid concentrations. **(A)** Baseline comparison of fecal butyric acid concentrations between Group A (control group) and Group B (intervention group). **(B)** Comparison of fecal butyric acid concentrations between Group A (control group) and Group B (intervention group) at 3 months. **(C)** Within-group comparison of fecal butyric acid concentrations in Group B (intervention group) between baseline and 3 months.

Quantitative analysis of seven fecal SCFAs (acetic acid, propionic acid, butyric acid, isobutyric acid, valeric acid, isovaleric acid, and hexanoic acid) revealed no significant differences between groups or across time points. Butyrate is shown for visualization (Figure 10), with the remaining SCFAs showing no statistically significant changes.

#### 3.4.2 Screening of differential metabolites

**Figure 11:**
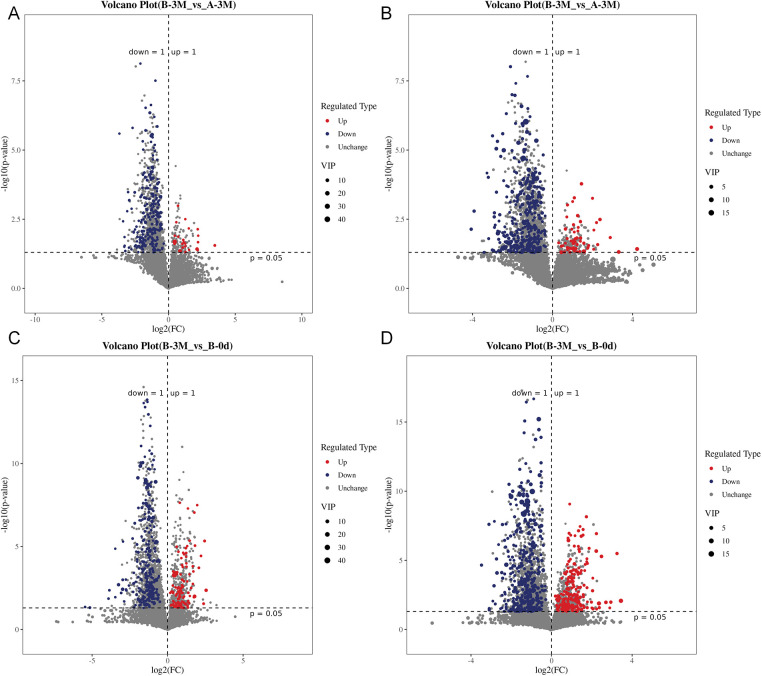
Volcano plots of differential metabolites identified in negative (neg) and positive (pos) ionization modes. **(A)** Comparison of metabolites between Group B (intervention group) and Group A (control group) at 3 months in neg mode. **(B)** Comparison of metabolites between Group B (intervention group) and Group A (control group) at 3 months in pos mode. **(C)** Within-group comparison of metabolites in Group B between baseline and 3 months (B-3M vs. B-0d) in neg mode. **(D)** Within-group comparison of metabolites in Group B between baseline and 3 months (B-3M vs. B-0d) in pos mode.

Differential metabolite analysis revealed that metabolic alterations were primarily associated with the intervention, with post-intervention between-group and within-intervention comparisons identifying more differential metabolites and higher statistical significance than the baseline between-group comparison. Consistent with these findings, volcano plots showed a broader distribution of differential metabolites along the log_2_(FC) axis and an overall upward shift along the −log_10_(P) axis, with downregulated metabolites (log_2_FC < 0) predominating, a pattern consistently observed in both positive and negative ionization modes (Figure 11).

#### 3.4.3 KEGG enrichment analysis

**Figure 12:**
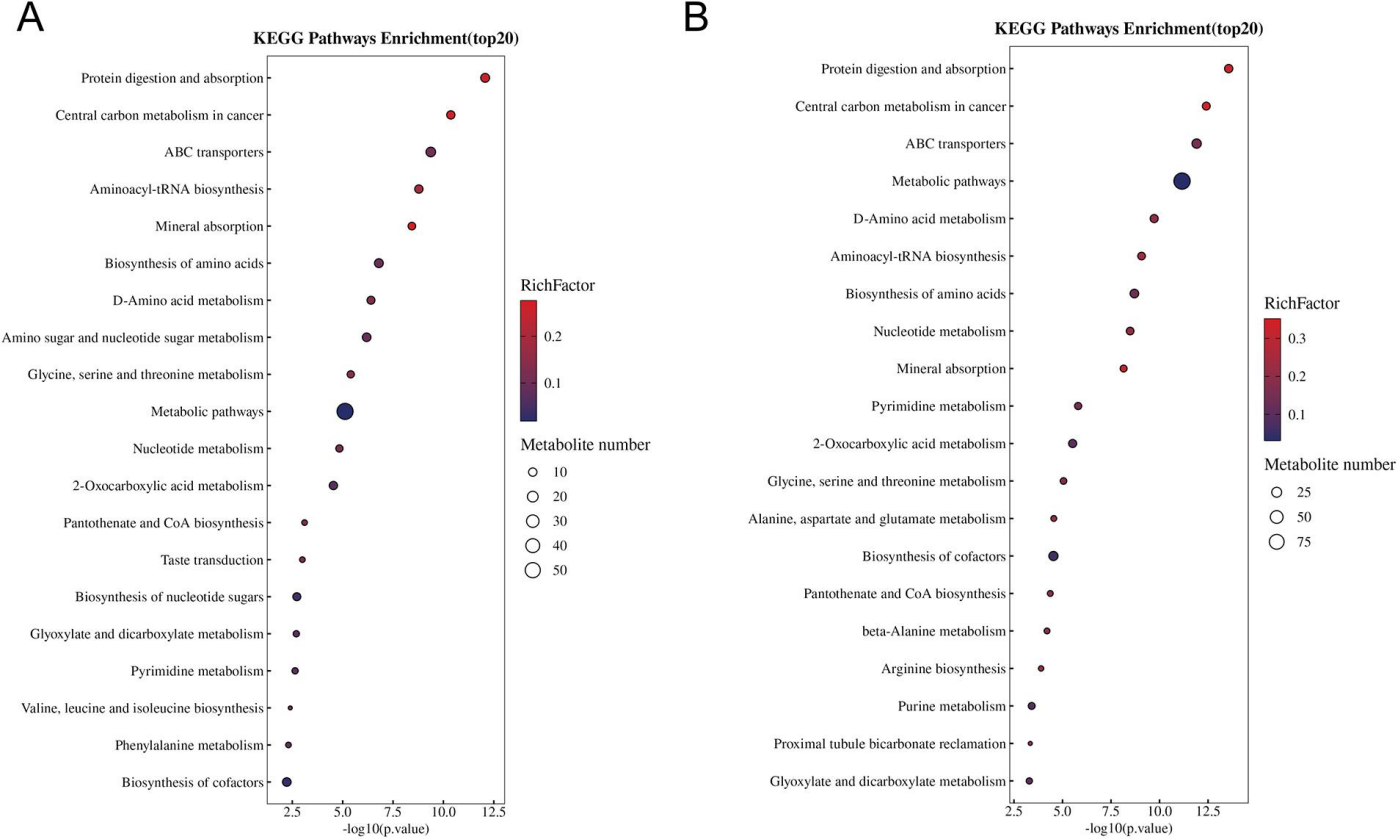
KEGG pathway enrichment analysis of differential metabolites. **(A)** KEGG pathway enrichment of differential metabolites between Group A (control group) and Group B (intervention group) at 3 months. **(B)** KEGG pathway enrichment of differential metabolites within Group B (intervention group) between baseline and 3 months.

KEGG pathway enrichment analysis revealed that differential metabolites identified post-intervention were significantly enriched in multiple metabolic pathways (Figure 12). Overall, the enriched pathways were predominantly associated with energy metabolism, integrated metabolic pathways, and substance transport processes, each showing clear statistical significance.

Among the significantly enriched pathways, protein digestion and absorption, central carbon metabolism in cancer, and ABC transporters exhibited the highest −log_10_(p) values and enrichment factors. Pathways closely linked to energy production and metabolic coordination—such as metabolic pathways and pantothenate and CoA biosynthesis—were also statistically significant.

In addition, differential metabolites were involved in pathways related to amino acid metabolism and nucleotide metabolism.

### 3.5 Integrative analysis of the gut microbiome and metabolome

#### 3.5.1 Core associations and drivers

**Figure 13:**
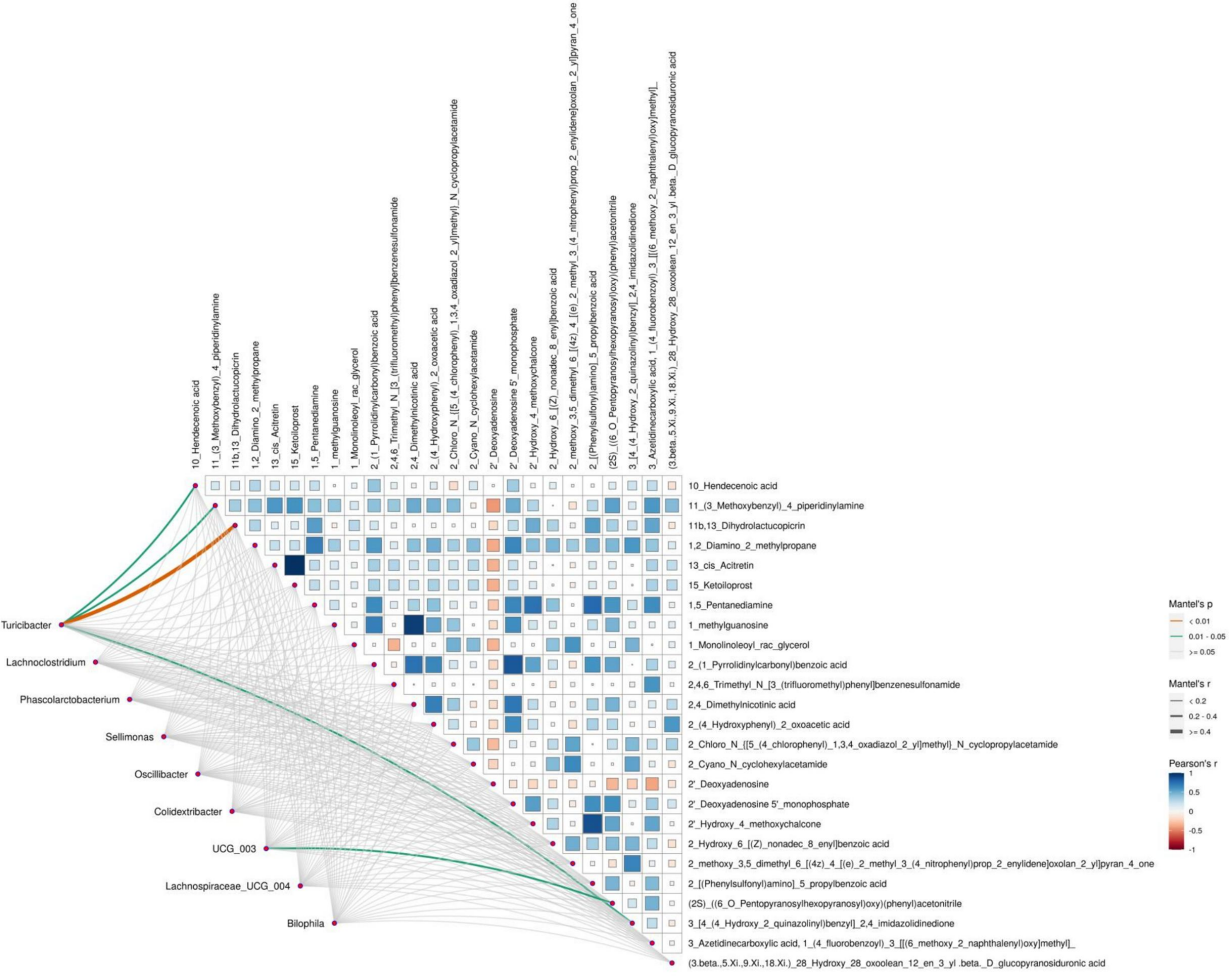
Mantel correlations between gut microbiota and metabolites based on the joint A/B dataset at 3 months.

An integrative analysis combining Mantel testing, RDA, and correlation network analysis revealed consistent associations between gut microbial composition and differential metabolites in the between-group comparison after 3 months of intervention. Mantel testing demonstrated significant associations between specific gut bacterial genera and the differential metabolite profile (*p* < 0.05; Figure 13). Among these, *Turicibacter* showed significant correlations with several differential metabolites, including 10-Hendecenoic acid, 11-(3-Methoxybenzyl)-4-piperidinylamine, and 11b,13-Dihydrolactucopicrin, and emerged as one of the key nodes within the association network.

**Figure 14:**
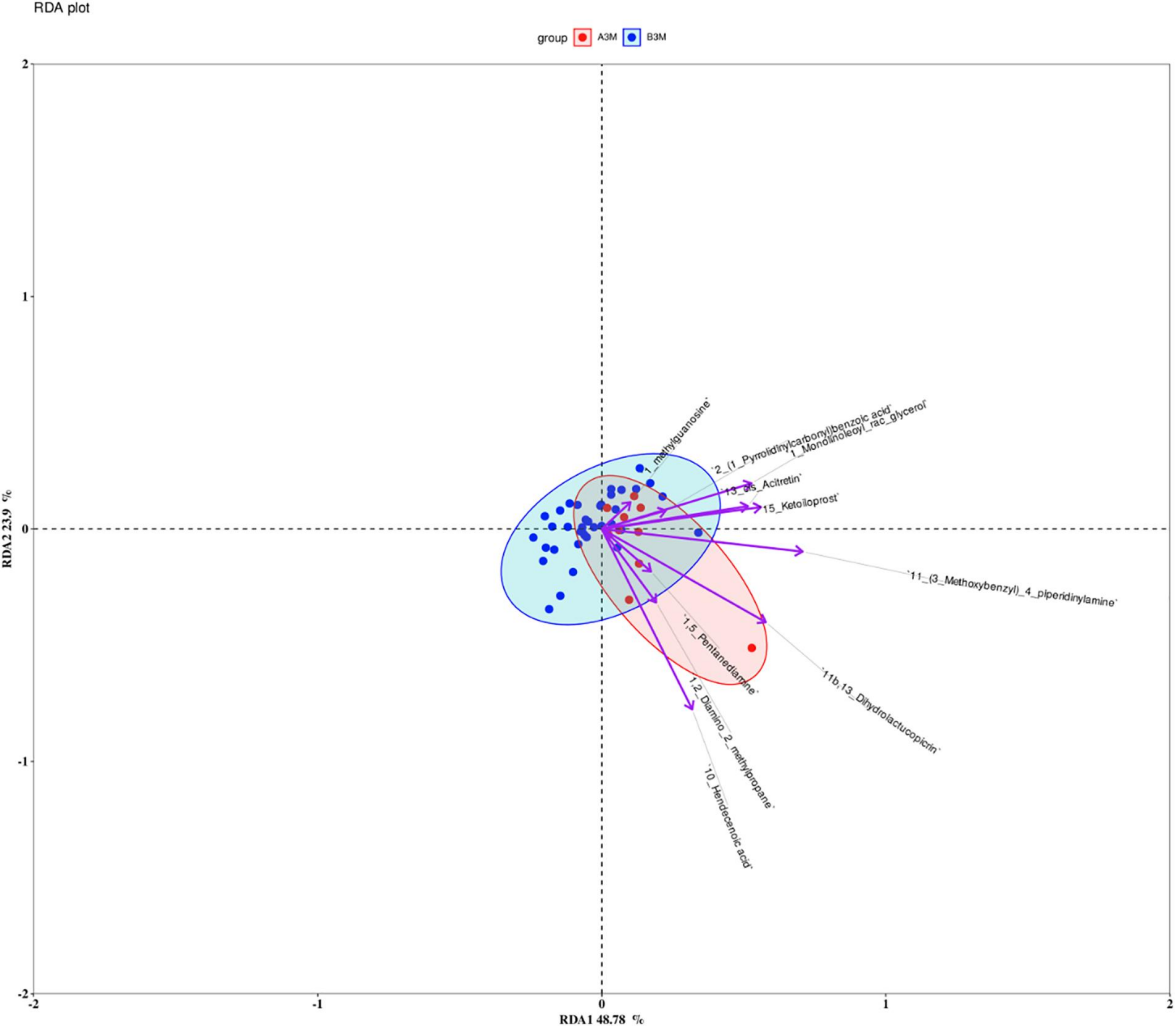
RDA ordination showing microbiota–metabolite associations based on the joint A/B dataset at 3 months.

RDA further characterized the relationship between metabolite variation and microbial community structure. The first two RDA axes explained 48.78% and 23.39% of the variance in microbial composition, respectively, accounting for the majority of the explained community variation (Figure 14). In the RDA biplot, the above metabolites were represented by vectors with pronounced lengths and clear directionality, indicating a strong contribution to the ordination structure and a substantial association with microbial community variation.

#### 3.5.2 Network analysis

**Figure 15:**
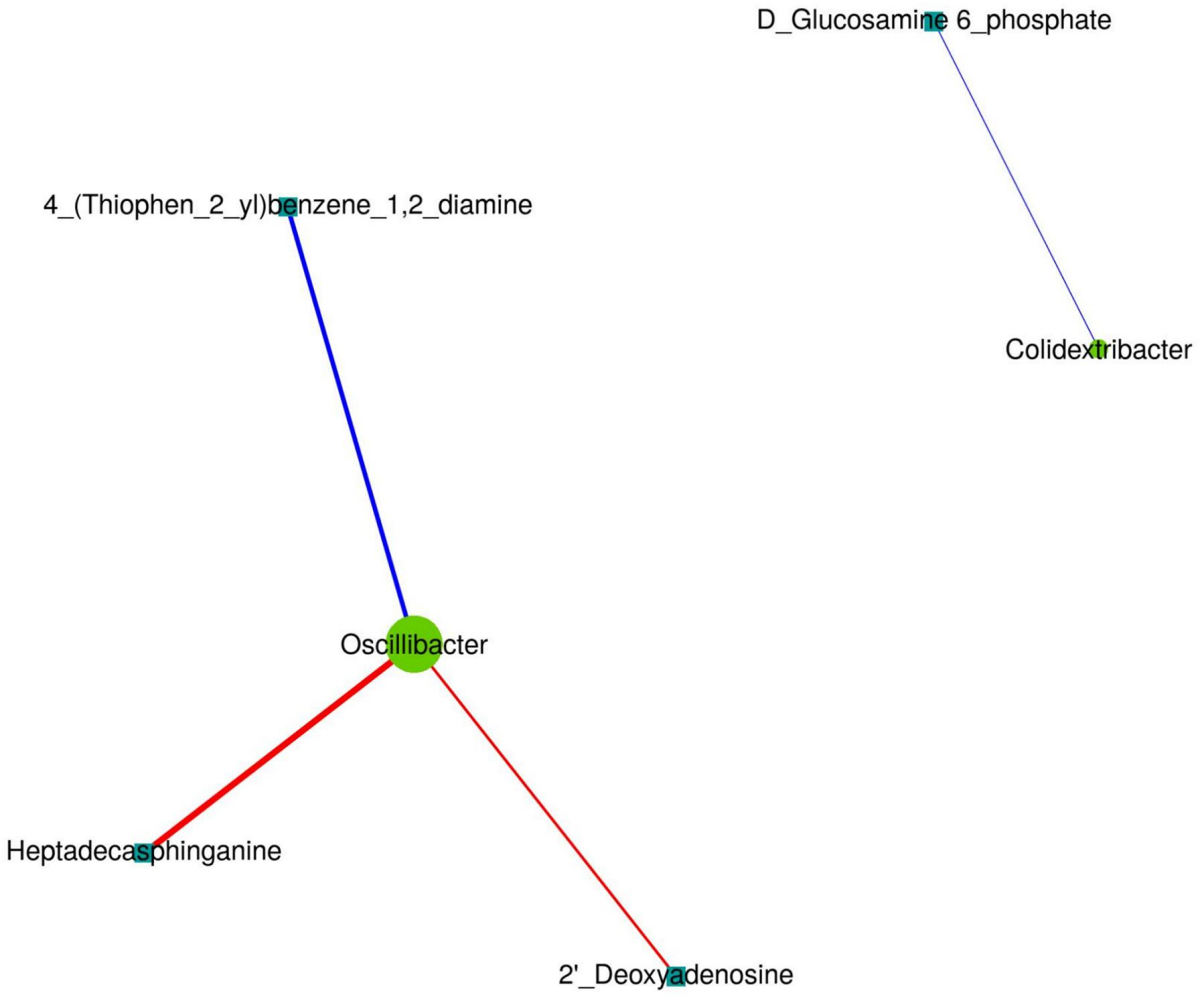
Network representation showing microbiota–metabolite associations based on the joint A/B dataset at 3 months.

Network analysis characterized the association structure between differentially abundant bacterial genera and differential metabolites (Figure 15). Among these, *Oscillibacter* exhibited a higher degree of connectivity within the network and showed significant associations with multiple differential metabolites: it was positively correlated with Heptadecasphinganine and 2′-Deoxyadenosine, and negatively correlated with 4-(Thiophen-2-yl)benzene-1,2-diamine. In addition, *Colidextribacter* was negatively correlated with D-Glucosamine-6-phosphate.

## 4 Discussion

A central finding of this study is that supplementation with lactoferrin combined with Bifidobacterium animalis subsp. lactis BB-12 in healthy children was associated with clinical improvement alongside concurrent alterations in gut microbial structure and functional profiles, suggesting that this combined intervention may contribute to the maintenance of host immune homeostasis through modulation of the gut microbiome.

In the present study, the combined intervention was associated with attenuation of symptoms and a reduced disease burden among children with RTIs, rather than a significant reduction in RTI incidence. Similar patterns have been reported in some clinical trials, in which nutritional or probiotic interventions primarily influenced disease severity and duration rather than infection occurrence (11). This discrepancy across studies may be attributable to differences in study design, outcome definitions, and statistical power, and is biologically plausible given that such interventions are more likely to modulate host immune responses and inflammatory tone than to prevent pathogen exposure *per se*. Moreover, the study population comprised healthy, non–high-risk children with a low baseline susceptibility to RTIs, in whom RTI occurrence is largely influenced by environmental exposure and epidemiological factors. Under these conditions, and given the limited sample size and follow-up duration, RTI incidence is a relatively insensitive endpoint for detecting intervention-related effects.

Safety follow-up indicated a lower incidence of dyspepsia in the intervention group than in the control group. Although not a prespecified endpoint, this exploratory signal suggests a potential gastrointestinal benefit of combined lactoferrin and probiotic supplementation in healthy children, consistent with prior randomized evidence (12, 13), with its clinical relevance best clarified in studies specifically designed around gastrointestinal outcomes.

To explore the biological mechanisms mediating the observed clinical benefits of the combined intervention, we further interrogated alterations in gut microbial composition. In this analysis, LEfSe revealed baseline differences between the two groups across multiple taxonomic levels prior to intervention. Such microbial imbalances are not uncommon in pediatric microbiome research and may arise from interindividual variations in early-life environmental exposures, dietary habits, and developmental trajectories of the gut microbiota. Notably, these baseline differences were predominantly observed in conditionally pathogenic or inflammation-associated taxa rather than core beneficial genera, providing important context for interpreting subsequent intervention-related effects.

At the genus level, LEfSe and STAMP analyses showed concordant patterns. The control group was characterized by a higher relative abundance of *Turicibacter*, a genus previously reported to be enriched in settings of immune hyperactivation, such as immune-related adverse events during immune checkpoint inhibitor therapy (14). In contrast, the intervention group exhibited increased abundances of SCFAs-associated genera, including *Lachnoclostridium* and *Oscillibacter*, suggesting a shift toward microbial communities with greater potential for SCFAs-related metabolic functions. At a finer taxonomic resolution, STAMP further revealed enrichment of taxa within the *Clostridium leptum* group—commonly linked to butyrate-related metabolism—in the intervention group, whereas *Rothia mucilaginosa*—often regarded as a conditionally pathogenic species or a marker of dysbiosis—was more abundant in the control group. These finer-resolution findings further reinforce the direction of microbiota shifts identified at the genus level.

During the intervention, distinct temporal trajectories of the gut microbiota were observed. Compared with the greater temporal variability observed in the control group, the intervention group exhibited a more stable overall community structure, with smoother longitudinal changes in the abundance of dominant taxa. This stability was most apparent in key functional groups: the well-recognized butyrate associated genus *Faecalibacterium* remained relatively stable and showed a gradual increase in the intervention group, whereas it declined markedly early during follow-up in the control group with only partial recovery thereafter. In contrast, the potentially pathogenic or opportunistic genus *Escherichia-Shigella* increased progressively in the control group but showed no clear time-dependent accumulation in the intervention group. These patterns are consistent with an intervention-associated stabilization of a community structure relatively enriched in SCFA-producing taxa, alongside a restraint of potentially unfavorable microbial expansion, suggesting a more balanced gut microbial profile.

Against this background, *Bifidobacterium* in the intervention group increased early after supplementation but showed a partial decline by 3 months. This fluctuation does not necessarily indicate attenuation of the intervention effect but is more likely to reflect coordinated regulatory dynamics within the gut microbiota. Previous studies have reported reciprocal patterns between *Bifidobacterium* and *Bacteroides* (15)*,* driven by niche partitioning and competition for shared substrates in the intestinal ecosystem (16). In the intervention group, Bacteroides showed a sustained increase over time and may have partially compensated for functional roles associated with Bifidobacterium. Both taxa are recognized as key symbionts in the pediatric gut, and their overall abundance and temporal stability in the intervention group exceeded those observed in the control group. These patterns indicate that microbiota changes induced by the combined intervention are not characterized by persistent shifts in a single genus, but rather by coordinated adjustments among functionally related taxa and broader ecological restructuring.

Building on the observed taxonomic differences and temporal patterns, comparisons based on PD-whole tree and weighted UniFrac revealed statistically significant differences after the intervention, indicating that the impact of the intervention extended beyond stochastic fluctuations of individual taxa to encompass coordinated shifts in phylogenetic structure and dominant community composition. By contrast, no significant changes were detected in other diversity indices, suggesting that the combined intervention did not induce broad ecological disruption and instead exerted targeted modulation of functionally relevant, high-contribution taxa while preserving overall community stability.

To further translate these compositional shifts into functional implications, we next examined microbial metabolic pathways and microbe-metabolite interactions. KEGG pathway enrichment analysis identified amino acid, nucleotide, and energy metabolism as the primary categories of differential pathways, pointing to a system-level reorganization of microbial metabolic networks following the intervention. Against this metabolic background, integrated microbiome-metabolome analysis showed that microbe-metabolite covariance in the control group was largely driven by metabolites related to fatty acid oxidation and energy metabolism, whereas this dominance was substantially reduced after intervention. In line with these findings, although microbial compositional and functional analyses consistently pointed to altered SCFAs-related metabolic potential, no significant differences were detected in fecal SCFAs concentrations. Taken together, these results suggest that the intervention was associated not with increased accumulation of terminal energy metabolites, but with a redistribution of metabolic associations across multiple pathways, indicative of a more balanced metabolic profile rather than a shift toward a single dominant energ*y* axis.

These findings support a plausible mechanistic framework in which the combined lactoferrin–probiotic intervention does not act through enhancement of a single metabolic pathway, but instead operates via coordinated remodeling of gut microbial structure and metabolic regulation, thereby improving the intestinal microenvironment while maintaining metabolic homeostasis. Such microbiota- and metabolism-related changes may collectively contribute to the regulation of respiratory mucosal defense. Although the present study does not permit direct delineation of specific molecular mechanisms, it provides a clear entry point for future investigations targeting key nodes along the microbiota–metabolite–immune axis.

While this study offers valuable insights, several limitations should be taken into account when interpreting the findings. The age range of the study population (0–6 years) is associated with considerable variability in gut microbiome composition and responses to exogenous probiotics, particularly across different age groups. This variation in immune system development and microbiota stability could influence the effectiveness of the intervention. Additionally, although changes in gastrointestinal symptoms were observed, it was not possible to definitively determine whether these symptoms were directly linked to the study medication. Other potential confounding factors, such as environmental and dietary influences, could have played a role.

The combined intervention design also posed a challenge, as it did not allow for the separate evaluation of the individual effects of probiotics, lactoferrin, and galactooligosaccharides (GOS). As a result, distinguishing the independent contributions of each component to the observed outcomes remains difficult. Interactions between these components may have contributed to the overall effect, making it harder to assess the specific impact of each. Additionally, despite efforts to control intervention conditions, participants' daily food and supplement intake may still have affected their gut microbiomes, and these factors were not fully controlled in the study, limiting our ability to account for their influence on the results.

The study's design as a single-center trial with a relatively small sample size and short follow-up period also limits the ability to assess long-term effects. Although we used methods like 16S rRNA-based functional prediction, untargeted metabolomics, and targeted quantification of short-chain fatty acids, these techniques have inherent limitations in providing a detailed mechanistic understanding. Future studies incorporating larger cohorts, longer follow-up, and metagenomic or more comprehensive targeted metabolomic analyses are needed to further validate and extend these findings.

## 5 Conclusion

Overall, this study indicates that combined lactoferrin and *Bifidobacterium animalis* subsp. *lactis* BB-12 intervention in healthy children is associated with alterations in gut microbiota structure and functional metabolic regulation, which may in turn contribute to modestly improved RTI outcomes. By integrating microbiome and metabolomic analyses, this study provides new evidence supporting the potential value of combined microecological interventions for the prevention of childhood infections. However, the long-term effects of this intervention, as well as the optimal target populations and application strategies, remain to be clarified in larger-scale studies with extended follow-up.

## Data Availability

The original contributions presented in the study are included in the article/supplementary material, further inquiries can be directed to the corresponding authors.
